# Hypoxic preconditioning effect on stromal cells derived factor-1 and C-X-C chemokine receptor type 4 expression in Wistar rat’s (*Rattus norvegicus*) bone marrow mesenchymal stem cells (*in vitro* study)

**DOI:** 10.14202/vetworld.2018.965-970

**Published:** 2018-07-19

**Authors:** Sri Wigati Mardi Mulyani, Diah Savitri Ernawati, Eha Renwi Astuti, Fedik Abdul Rantam

**Affiliations:** 1 Department of Dentomaxillofacial Radiology, Faculty of Dental Medicine, Universitas Airlangga, Surabaya, Indonesia; 2 Department of Oral Medicine, Faculty of Dental Medicine, Universitas Airlangga, Surabaya, Indonesia; 3 Stem Cell Research Center and Development, Airlangga University Surabaya, Indonesia; 4 Lab of Virology and Immunology, Department of Microbiology, Faculty of Veterinary Medicine, Universitas Airlangga, Surabaya, Indonesia

**Keywords:** bone marrow stem cells, C-X-C chemokine receptor type 4, hypoxic preconditioning, mesenchymal stem cells, stromal cells derived factor-1

## Abstract

**Aim:**

To examine the effect of hypoxic preconditions on the ability of bone marrow stem cells culture mediated expression C-X-C chemokine receptor type 4 (CXCR4) and stromal cells derived factor-1 (SDF-1) *in vitro*.

**Materials and Methods:**

Bone marrow mesenchymal stem cells (BMSCs) were derived from 12 femurs of 200 g Wistar male rats. The animals were euthanized before BMSCs isolation. BMSCs were divided into two groups, control group: Normoxic condition 21% O_2_ and treatment group: Hypoxic condition 1% O_2_. The characterization of BMSCs was analyzed using flow cytometry by cluster differentiation 34 and cluster differentiation 105. The expression of CXCR4 and SDF-1 measured using immunocytochemistry immunofluorescence label after 48-h incubation in a low-tension oxygen chamber with an internal atmosphere consisting of 95% N_2,_ 5% CO_2_, and 1% O_2_. All data were subjected to a normality test and then analyzed using t-test statistic (p<0.05).

**Results:**

The characterization of bone marrow stem cells showed positive cluster differentiation 34 and cluster differentiation 105. A hypoxic precondition (1% O_2_) in culture increases CXCR4 (p=0.000) and SDF-1 expression than normoxic conditions (p=0.000) (p<0.05).

**Conclusion:**

Hypoxic preconditioning with 1% O_2_ increase CXCR4 and SDF1 expression.

## Introduction

Mesenchymal stem cells (MSCs) constitute a population of adult stem cells that can transdifferentiate not only into types of mesodermal lineage cells but also into multilineage cell types [1]. Preliminary studies have shown that MSCs are capable of producing a range of cytokines and growth factors such asbasic fibroblast growth factor and vascular endothelial growth factor (VEGF). In addition, there is another factor that significantly influences the success of therapy by inducing stem cells to migrate into defective areas. The other factors that can mediate such migration are stromal derived-cell factor 1 (SDF1) and C-X-C chemokine receptor type 4 (CXCR4) [2].

SDF-1 is a small molecule of chemokines which, by binding with a CXCR4 receptor, executes an important role in regulating the adhesion, expansion, migration, and homing of MSCs. A recent study indicates that CXCR4 and SDF-1 are highly expressed in bone marrow MSCs (BMSCs), but is lost on culturing at a high passage number. Under hypoxic conditions, a number of cytokines, chemokines including CXCR4 and SDF-1 expression can be re-established, thereby maintaining the efficacy of MSCs. The survival and proliferation of transplanted progenitor cells in tissue would require cell adaptation to the harsh, low oxygen tension environment [3]. The previous study demonstrated that hypoxia preconditioning can increase the ability of transplanted stem/progenitor cells to survive and proliferate ability *in vitro* [4].Hypoxia preconditioning mimics the situation that the cells will meet when they are transplanted into the tissue defect. Accordance to the previous study demonstrated thatHypoxia-inducible factor-1 alpha (HIF-1α), CXCR4, anti-apoptotic gene Bcl-2, p-Akt, SDF-1α, and VEGF expression were all elevated after hypoxia preconditioning and were further verified by increased anti-apoptosis and migration capacity *in vitro* and decreased apoptosis and better cardiac rescue potency *in vitro*. However, the mechanisms underlying the beneficial effects of hypoxia preconditioned progenitor cells remain incomplete [5].

The aim of this study was to examine the effect of hypoxic preconditions on MSCs culture to expression CXCR4 and SDF-1 *in vitro*.

## Materials and Methods

### Ethical approval

All animal studies were performed through a protocol approved by the Institutional Animal Care and Use Committee of Faculty of Veterinary Medicine, Universitas Airlangga and complied with the National Research Council’s guidelines ethical approval number: No 366-KE through the ethical seminar. The research was conducted at an experimental laboratory within the Stem Cell and Tissue Engineering Development Centre, Universitas Airlangga, Surabaya, Indonesia.

### Animal model

All animals were housed in polycarbonate cages, subjected to a 12-h light-dark cycle at the constant temperature of 23°C, and fed a standard pellet diet (expanded pellets, Stepfield, Witham, Essex, UK) with tap water *ad libitum*. The exploration of MSCs involved the use of 200 femurs from 12 Wistar male rats. The sampling technique of the BMSCs from the rat’s femurs was determined by Lemeshow’s method. The animals were euthanized before Bone Marrow isolation.

### BMSCs isolation

BMSCs isolation and culture according to Rantam *et al*.’s [6] method aspirate from femur bones produces sufficient marrow to be cultured. Aspirate of bone marrow was attached to a 15 ml Heparin tube (Sigma-Aldrich^®^, USA) previously filled with 3 ml α-minimum essential medium (MEM). Each aspirate was transferred to a 15 ml sterile tube with a blue cap and diluted with 1× phosphate buffer saline (PBS) (Sigma-Aldrich^®^, USA) sterile to a total volume of 8 ml. Each tube was then rinsed twice with 5 ml × PBS, and the contents were combined with an aspirate solution. In every case, the aspirate was placed in a Ficoll (Sigma-Aldrich^®^, USA) temperature chamber in a separate 15 ml tube. Furthermore, each aspirate was coated with Ficoll before being centrifuged (Sorvall™ MX Series Floor Model Micro-Ultracentrifuge, Thermo Fisher, USA) at 1600 rpm for 15 min at room temperature. After centrifugation, the collection was effected at the “buffy coat” location on the surface of Ficoll-PBS using a sterile Pasteur pipette and placed in a 15 ml tube (Sigma-Aldrich^®^, USA).

Each sample was diluted with 1 × PBS to a total volume of 15 ml, with the tube being turned 3-5 times as a means of achieving an even mix. At the next stage, centrifugation was undertaken for 10 min at a speed of 1600 rpm before the supernatant and cells suspended in 6 ml of cell culture medium (CCM) were removed before heating. Having placed between 5 cm^2^ and 10 cm^2^ of cells on the plate, they were incubated at 37°C at 5% CO_2_ moisture and allowed to attach to the cell for 18-24 h. Approximately 24 h later, the media and cells not attached were disposed of. 5 ml 1 × PBS was then added before being heated in the culture, shaken well and used to cover the surface area. It was then disposed of with 1 × PBS before the washing process being repeated twice.

Ten minutes later, 10 ml of fresh CCM was added to the dish before it was returned to the incubator. The cells were incubated at 37°C and 5-10% CO_2_ moisture with the culture being observed daily using an inverted microscope. Every 3 days, the media were removed and the cells rinsed with 5 ml or 10 ml of 1 × PBS before heating. The PBS was subsequently discarded and the dish filled with 10ml of fresh CCM. This process was continued until the concentration of confluent cells reached between 60% and 80%. If the cell developed in the cell plant, the latter had to be balanced in the incubator at 5% CO_2_ moisture and 37°C for 48 h before use.

### BMSCs identification surface markers by flow cytometry

BMCs were harvested by centrifugation at the end of coculture and made in single cells before flow cytometry analysis. The cells are then incubated in test tubes or microtiter plates with unlabeled or fluorescently conjugated antibodies and analyzed through flow cytometry (Becton Dickson FACSVerse, San Diego, USA). MSCs were trypsinized for identification of a number of MSC surface markers, approximately 2×10^5^ cells per sample were washed twice with PBS. The antibodies for surface markers were anti-CD34 - Allophycocyanin (Cat no. I345804) anti-CD105 Fluorescein Isothiocyanate (FITC) (Cat. No 561443) (Becton Dickson Pharmingen, San Diego, USA).

### BMSCs culture

Bone marrow samples were dissolved in three equal volumes of the MSC growing medium and distributed uniformly across 10 cm culture dishes. Each dish produced 10 ml of diluted aspirate. Stored in an incubator (Thermo Scientific Heraeus, USA) with 5% CO_2_ and cultured at a constant temperature of 37°C for 4-5 days. The medium was replaced every 3-4 days, and the contamination of red cells and unreplicated, unattached cells were eventually diluted and rinsed to eradicate it.

Small MSC colonies of fibroblast cells were attached and perceptible within 5-7 days. After 12-14 days, small colonies could easily be detected. In this condition, cells were rinsed with serum-free α-MEM and subculture. 5 ml of 0.05% trypsin was added and, after a few minutes, cells began to be dispatched from the substrate. Dial the observations under a microscope (JEOL, JSMT1000, Scanning Microscope, Japan). The trypsin/ethylenediaminetetraacetic acid (Sigma-Aldrich^®^, USA) solution could be carefully aspirated and removed as long as the full cells had not been delivered. If the removal of cells from the substrate had begun, a fresh growing medium containing fetal bovine serum was added which eliminated trypsin activity. The MSCs were then flushed from the surface with a flask grower incorporating a pipette and divided into two dishes at this stage of low concentration and then rapidly expanded. Each dish was filled with 10 ml of cell susceptibility before being placed in 5% CO2 incubator. BMSCs were divided into two groups, control group: Normoxic condition 21% O_2_ and treatment group: hypoxic condition 1% O_2_.

### Hypoxia preconditioning

Hypoxia was achieved by placing the cells in a Modular Incubator Chamber (Billups-Rothenberg; Del Mar, CA) according to the manufacturer’s instructions. After a brief time spent in the chamber, the cells were flushed with a mixture of 0.1% O_2_, 5% CO_2_, and 94.9% N_2_ for 5 min. The chamber was then closed and the cells incubated at 37°C for various lengths of time. Next, the cells were cultured under normoxic or hypoxic conditions for 3 h, 6 h, 12 h, and 24 h.

### Identification of CXCR4 and SDF-1 expression by immunofluorescence

Trypsin was added and centrifuged at 1600 rpm for 5 min. The pellets were added to 1 ml of α-MEM growth medium (Sigma-Aldrich^®^, USA), suspended and grown on a special glass of 20 μl. The object glass was placed in a box containing wet paper and then incubated at 37°C for 1 h before being washed 4 times with PBS and dried. Anti-CXCR4 (Cat No. MAB172) and anti-SDF-1 (Cat No. MAB350) monoclonal antibodies (Sigma Aldrich^®^, USA) were added to each sample and then incubated at 37°C for 45 min. After that, the PBS washing and drying processes were repeated. At the next immunofluorescence, FITC labeled examination on glass object with 50% glycerin dropped above the glass object and viewed the results with a fluorescent microscope (Automated Fluorescence Microscope, BX63, Olympus^®^, USA).

### Statistical analysis

Data in normal distribution were analyzed using a t-test. Statistical analysis was analyzed using of Statistical Package for the Social Sciences (SPSS) 17.0 software for windows 8.1 by SPSS Inc., Chicago, United States.

## Results

**Figure-1:**
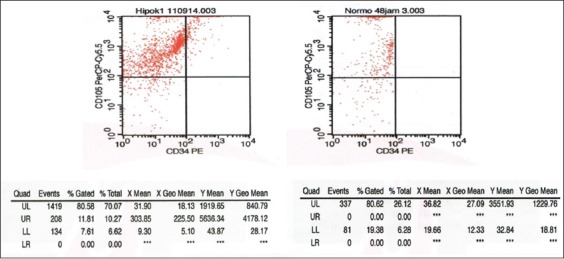
Phenotypic characterization of bone marrow mesenchymal stem cells identified by flow cytometry examination.

**Figure-2:**
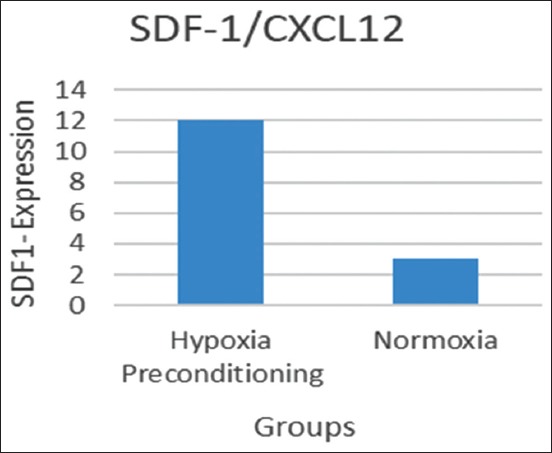
The mean of stromal cells derived factor-1/C-X-C motif chemokine 12 expressions in bone marrow mesenchymal stem cells cultured in both hypoxia preconditioning and normoxia group.

**Figure-3:**
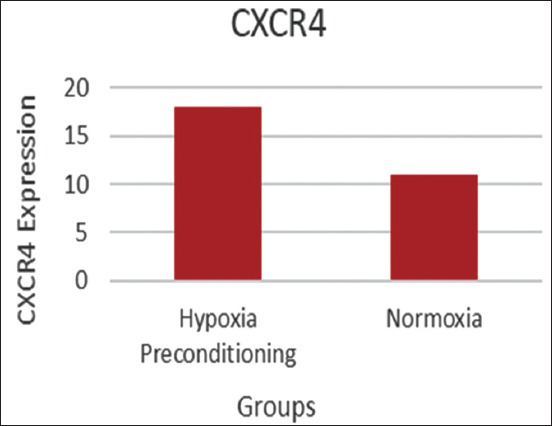
The mean of C-X-C chemokine receptor type 4 expression in mesenchymal stem cells cultured in both hypoxia preconditioning and normoxia group with ICC examination.

Flow cytometry analysis of BMSC phenotypes and BMSC cultures treated with hypoxic preconditioning was expressed more strongly at CD105 (70.07%) than the normoxic condition (26.12%) and CD34 expression negatively in both conditions (Figure-1). The mean of SDF-1 and CXCR4 expression significantly expressed in hypoxic preconditioning group than normoxic. The t-test result indicated a significant difference (p<0.05) between the SDF1/C-X-C motif chemokine 12 (CXCL12) expression between groups (Figure-2 and Table-1). The t-test result demonstrated that there was a significant difference (p<0.05) between CXCR4 expressions of the hypoxic preconditioning group and the normoxic group (Figure-3 and Table-2).

**Table-1:** T-test result of SDF-1 expression in BMSCs culture hypoxic preconditioning and normoxic groups.

Group	SDF-1	Significant

	Mean±SD	
Hypoxic	11.677±2.447	p=0.000*
Normoxic	3.517±0.969	

* Significant (p<0.05). SDF-1=Stromal cells derived factor-1, BMSCs=Bone marrow mesenchymal stem cells, SD=Standard deviation

**Table-2:** T-test result of CXCR4 expression in BMSCs culture hypoxic preconditioning and normoxic groups.

Group	Mean±SD	P
Hypoxic	18.200±5.596	0.000*
Normoxic	10.750±5.748	

* Significant (p<0.05). BMSCs=Bone marrow mesenchymal stem cells, SD=Standard deviation, CXCR4=C-X-C chemokine receptor type 4

**Figure-4:**
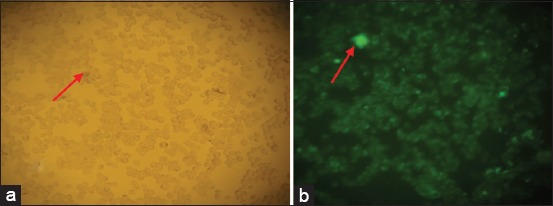
The expression of stromal cells derived factor-1 at 4^th^ passage in a normoxic group with 100× magnification. (a) Mesenchymal stem cells without filter; (b) with fluorescent filter (NikkonH600L Microscope; digital camera DS Fi2 300 megapixel).

**Figure-5:**
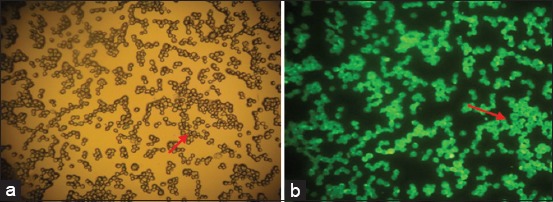
The expression of stromal cells derived factor-1 at 4^th^ passage in hypoxic preconditioning group with 100×. (a): Mesenchymal stem cells without filter; (b) with fluorescence filter (NikkonH600L Microscope; digital camera DS Fi2 300 megapixel).

**Figure-6:**
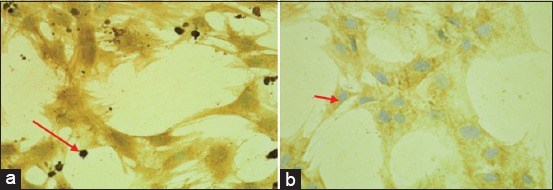
C-X-C chemokine receptor type 4 expression (chocolate chromogen) on the mesenchymal stem cell hypoxic preconditioning group (a) showed strong expression, whereas the normoxic group demonstrated weak expression (Slide B) with immunocytochemistry with 400× (NikkonH600L Microscope; digital camera DS Fi2 300 megapixel).

The immunofluorescence of SDF-1 expression result showed the normoxic condition (O_2_ 21%) to be weakly expressed but more strongly so after a 48-h hypoxic precondition (O_2_ 1%) (Figures-4 and 5). The immunocytochemical of CXCR4 expression result showed weakly expressed in normoxic condition (O_2_ 21%) but strongly expressed (chocolate chromogen) in hypoxic condition (O_2_ 1%) (Figure-6).

## Discussion

The important factor in cell-based regenerative therapy is the ability of stem cells to migrate to the defect areas through trafficking and successful engraftment when stem cells are transplanted. This is strongly influenced by the CXCR4 chemokine receptor bond with the SDF-1 ligand chemokine. The loss of this chemokine receptor during expansion *in vitro* culture will decrease the regenerative ability of stem cells [7]. The culture environment affects cell aging and expression of the chemokine marker that plays an important role in migratory cells and engraftment when MSCs are transplanted. These problems can be minimized by modifying the microenvironment in stem cell culture by providing precondition of hypoxia with the oxygen concentration in accordance with its niche environment (O_2_ 1-3%) [8].

MSCs are known to have some beneficial properties such as being found in BMSCs [9]. Previous studies reported the ability of MSCs to secrete cytokines, chemokine, and growth factors in cultured cells which play an important role in the regeneration process such as SDF-1, CXCR4, VEGF, fibroblast growth factor, and insulin-like growth factor. These factors contribute to the migration cell process, survival cells, angiogenesis, cell proliferation, and differentiation that relates to tissue repair and regeneration [10].

The decreased potential of MSCs *in vitro* may be due to cell culture conditions and the total subcultures performed. Sohni and Verfaillie [11] revealed that the higher the number of passages made in a stem cell culture will decrease the potential for differentiation, viability, and effectiveness. Previous studies reported that term culture (40 days) will lead to loss of chemokine receptor expression followed by decreased expression of specific surface receptors (CD105 and CD90). Therefore, it can be concluded that MSCs require microenvironment to maintain their viability and plasticity [12]). Several studies suggest that hypoxic preconditioning will activate some transcription factors in the nucleus such as HIF-1α, nuclear factor kappa β, Wnt4, and miR210, where these will also interact with paracrine factors such as MEK, PI3K, Erk, and Akt that will increase the secretion of SDF-1 and CXCR4 expression [13].

One of the primary functions of the SDF1-CXCR4 is the trafficking regulation of MSC cells in homing in on the injury site [14]. The previous study demonstrated that MSC therapy to mice that produced a defect in their brains suggested that the migration process from MSCs to defective areas is probably mediated by chemokine and their receptors SDF1-CXCR4 through the mechanism of MSCs trafficking G-protein-coupled receptor signaling. SDF1-CXCR4 also plays a role in cellular retardation, proliferation, and differentiation mechanisms by MAPK/PI3K signaling pathway through increased expression of BCL2 and ERK [15].

In this study was to determine whether hypoxia preconditioning can improve the expression of chemokine receptors and ligand (CXCR4-SDF-1) in cells culture. BMSCs were taken from the femur of male Wistar rat and are cultured in hypoxic conditions (O_2_ 1%) at the 4^th^ passage and compared with normoxic condition (O_2_ 21%). The phenotypic characterization of MSCs using by flow cytometry in hypoxic condition showed strong expression of CD 105 compared to normoxic condition, the specific surface marker of MSCs and negative expression of CD 34 in both conditions, the specific marker of hematopoietic stem cells. It was assumed that the cell culture in hypoxic condition has purely isolated of MSCs than normoxic condition cell culture (Figure-1). The result of an examination on the effect of hypoxic precondition in the cell culture using immunofluorescence and immunocytochemical indicated strongly expressed of SDF-1 and CXCR4 after 48 h hypoxic precondition compared to the normoxic condition.

It was in accordance with Yellowley that revealed under hypoxic condition a number of cytokines, chemokines including CXCR4 and SDF-1 expression can be reestablished, so the efficacy of MSCs can be maintained. The expression of the transcription factor hypoxia-inducible factor-1, a-subunit (HIF-1a), may drive the upregulation of SDF-1/CXCL12 in hypoxic condition and ultimately regulate the homing of CXCR4 stem cells and progenitor cells. Under hypoxic conditions, the activity of PHD2 is reduced, and HIF-1a degradation is inhibited; HIF-1a accumulates and binds to its consensus sequence, the hypoxia-responsive element on HIF-1a target genes. 63 HIF-1a has been shown to induce the expression of SDF-1 and CXCR4. Finally, when MSCs transplanted can improve the ability of MSCs to migrate into defected tissues, proliferate, and differentiate into origin-like cells, and promote resident stem cells growth and proliferation.

## Conclusion

Hypoxic preconditioning 1% O_2_ can promote increasing CXCR4 and SDF1 expression that may play an important role to improve BMSCs migration into defect areas, proliferation, and differentiation into origin-like cells.

## Author’s Contributions

SWMM, DSE, ERA, FAR: Conception and design of the study. SWMM, DSE, ERA, FAR: Acquisition of data. SWMM, DSE, ERA, FAR: Analysis and interpretation of the data. SWMM, DSE, ERA, FAR: Drafting and revising the manuscript critically for important intellectual content. SWMM, DSE, ERA, FAR: All authors have read and approved the final manuscript.
